# Therapeutic Potential of Thiazolidinedione-8 as an Antibiofilm Agent against *Candida albicans*

**DOI:** 10.1371/journal.pone.0093225

**Published:** 2014-05-05

**Authors:** Mark Feldman, Abed Al-Quntar, Itzhak Polacheck, Michael Friedman, Doron Steinberg

**Affiliations:** 1 Biofilm Research Laboratory, Institute of Dental Sciences, Faculty of Dental Medicine, The Hebrew University of Jerusalem, Jerusalem, Israel; 2 Institute of Drug Research, School of Pharmacy, The Hebrew University of Jerusalem, Jerusalem, Israel; 3 Department of Clinical Microbiology and Infectious Diseases, Hadassah-Hebrew University Medical Center, Jerusalem, Israel; Ben-Gurion University of the Negev, Israel

## Abstract

*Candida albicans* is known as a commensal microorganism but it is also the most common fungal pathogen in humans, causing both mucosal and systemic infections. Biofilm-associated *C. albicans* infections present clinically important features due to their high levels of resistance to traditional antifungal agents. Quorum sensing is closely associated with biofilm formation and increasing fungal pathogenicity. We investigated the ability of the novel bacterial quorum sensing quencher thiazolidinedione-8 (S-8) to inhibit the formation of, and eradication of mature *C. albicans* biofilms. In addition, the capability of S-8 to alter fungal adhesion to mammalian cells was checked. S-8 exhibited specific antibiofilm and antiadhesion activities against *C. albicans*, at four- to eightfold lower concentrations than the minimum inhibitory concentration (MIC). Using fluorescence microscopy, we observed that S-8 dose-dependently reduces *C. albicans*–GFP binding to RAW macrophages. S-8 at sub-MICs also interfered with fungal morphogenesis by inhibiting the yeast-to-hyphal form transition. In addition, the tested agent strongly affected fungal cell wall characteristics by modulating its hydrophobicity. We evaluated the molecular mode of S-8 antibiofilm and antiadhesion activities using real-time RT-PCR. The expression levels of genes associated with biofilm formation, adhesion and filamentation, *HWP1, ALS3* and *EAP1*, respectively, were dose-dependently downregulated by S-8. Transcript levels of *UME6*, responsible for long-term hyphal maintenance, were also significantly decreased by the tested agent. Both signaling pathways of hyphal formation-cAMP-PKA and MAPK-were interrupted by S-8. Their upstream general regulator *RAS1* was markedly suppressed by S-8. In addition, the expression levels of MAPK cascade components *CST20, HST7* and *CPH1* were downregulated by S-8. Finally, transcriptional repressors of filament formation, *TUP1* and *NRG1*, were dramatically upregulated by our compound. Our results indicate that S-8 holds a novel antibiofilm therapeutic mean in the treatment and prevention of biofilm-associated *C. albicans* infections.

## Introduction

The yeast *Candida albicans* is found as a commensal microorganism in the digestive tract of mammals [1], as well as in the oral cavity of humans [2]. It is also the most common fungal pathogen in humans, causing both mucosal and systemic infections, particularly in immunocompromised individuals [3]. Several factors can predispose individuals to candidiasis, such as prolonged treatment with antibiotics, corticosteroids, diabetes mellitus, nutritional deficiencies, immunosuppressive diseases or hormone therapy [4]. Several virulence factors that contribute to the development of candidal infection have been identified. They include (i) adhesins that allow adhesion to human cells with subsequent invasion [5], (ii) the ability to form biofilm on human mucosa [5] and on artificial surfaces such as catheters [6], [7] and dental devices [8], [9], and (iii) the ability to switch from yeast to hyphal form [10]. Biofilm formation is an important factor in *C. albicans* pathogenesis [11]; it involves attachment, colonization and the development of a mature biofilm structure composed of yeast, pseudo- and true hyphae, and extracellular matrix [11]–[14]. Like bacterial biofilm, and in contrast to the planktonic form, fungal biofilm is highly resistant to antifungal drugs. This resistance is multifactorial and complex, involving: (i) limited drug penetration into the biofilm due to the high density of extracellular matrix, (ii) drug absorption or binding by the biofilm extracellular matrix, (iii) decreased growth rate, (iv) overexpression of genes involved in drug resistance, particularly those encoding efflux pumps, (v) and multidrug tolerance due to persistant cells [11], [13], [15], [16]The outcome of immobilized fungi in biofilm in terms of pathogenicity and drug resistance emphasizes the need for new antibiofilm agents that can inhibit biofilm formation or destroy preformed biofilm without affecting fungal viability.

It has been found that microorganisms actually exchange information between themselves. This cross-talk is termed quorum sensing (QS). QSis associated with biofilm formation and fungi's increased pathogenicity [17]. Indeed, fungal biofilm integrity is highly dependent on QS. Recently, two main QS regulators-farnesol and tyrosol-have been described in *C. albicans*. Farnesol regulates *C. albicans* morphology and inhibits biofilm formation [18], [19], whereas tyrosol is associated with increased biomass of *C. albicans* biofilms, probably by stimulating hyphal growth [20]. Thiazolidinediones (TZDs) have been proposed as potential QS inhibitors in *Vibrio harveyi* [21]. Several TZD derivatives have also been tested for their ability to affect *C. albicans* pathogenicity [16]. The aim of this study was to investigate the antibiofilm effect of the TZD derivative S-8 on the fungal pathogen *C. albicans*. In addition, the molecular mechanism governing S-8's antibiofilm activity was evaluated.

## Results

### Effect of S-8 on *C. albicans* viability and biofilm formation

**Figure 1:**
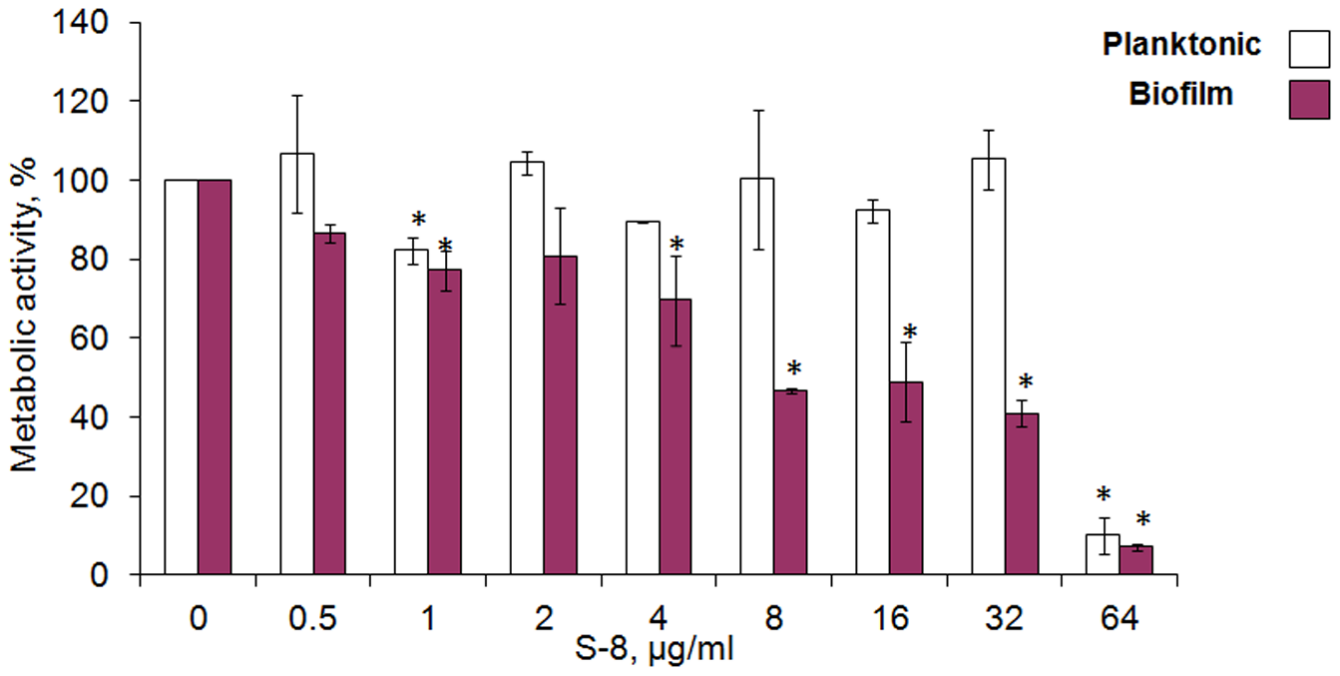
Effect of S-8 on *C. albicans* viability and biofilm formation. *C. albicans* cells were incubated with 0.5–64 µg/ml S-8 for 48 h. The metabolic activity of planktonic and biofilm cells was measured using the XTT reduction assay. The XTT values of the untreated control were set to 100%. The results are presented as means ± SD of three independent experiments. *Significantly lower than the untreated control (*P*<0.05).

Planktonic *C. albicans* viability, as measured by 2,3-bis(2-methoxy-4-nitro-5-sulfo-phenyl)-2H-tetrazolium-5-carboxanilide (XTT) reduction assay, was not significantly affected by S-8 up to 32 µg/ml, while the MIC value was 64 µg/ml. This concentration of S-8 reduced fungal metabolic activity by more than 90% (*P*<0.05) (Fig. 1). In contrast, S-8 dose-dependently decreased biofilm metabolic activity, and even at 8 µg/ml it inhibited biofilm formation by 50% (*P*<0.05), which was recorded as the minimal biofilm inhibitory concentration 50 (MBIC_50_) (Fig. 1). The *C. albicans* cell can reduce its metabolic activity as a protective mechanism under adverse conditions [22], [23]. Therefore, we studied the effects of S-8 on morphology and viability of the biofilm cells. Based on these results, in subsequent experiments we used S-8 at concentrations of up to 16 µg/ml, which is four times lower than the MIC, to prevent fungicidal effects of S-8 on the planktonic cells.

### S-8 affects fungal morphology in the biofilm

**Figure 2:**
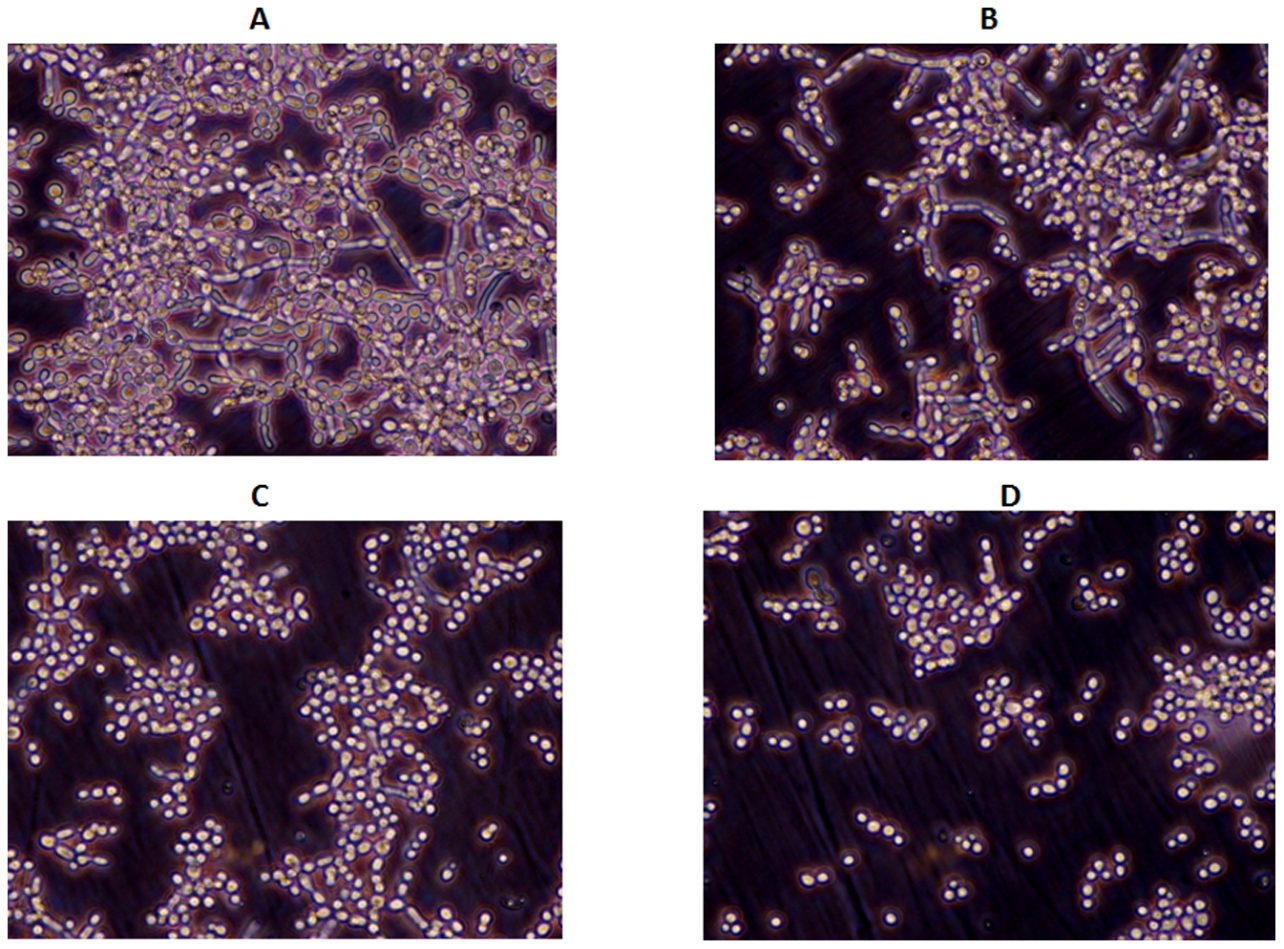
Effect of treatment with S-8 on *C. albicans* morphology. The morphology of *C. albicans* biofilms after treatment with S-8 was visualized using phase-contrast microscopy. A. Untreated control. B, C and D. Biofilms developed with 1, 4 and 16 µg/ml S-8, respectively. Magnification: 400X. At least four random fields were observed. Three independent experiments were performed.

Microscopic observation showed that in addition to decreasing metabolic activity in the biofilms, S-8 also inhibits the yeast-to-hyphal form transition, and reduces the length of existing filaments of adhered fungi. As shown in Fig. 2, untreated control biofilm (Fig. 2A) consisted of mostly branched hyphae and pseudohyphae, and only a few yeast cells. S-8 at 1 µg/ml increased the average number of yeast cells by 40% as compared to the untreated control, while shortening the average filament length by 1.3-fold (130 µm vs. 170 µm in the control) (Fig. 2B). Increasing the concentration of S-8 to 4 µg/ml dramatically reduced the average length of filaments adhering to the surface by 8.5-fold (20 µm vs. 170 µm in the control), while increasing the average count of the yeast form by 5.3-fold as compared to the untreated control (Fig. 2C). Finally, the highest tested dose of S-8 (16 µg/ml) resulted in almost total disappearance of the hyphal form with mostly yeast cells observed (Fig. 2D). This increase in S-8 concentration resulted in a sparse, loosely attached monolayer of *C. albicans* yeast cells, indicating disruption of the yeast-to-hyphal form transition, without causing cell death. Moreover, mycelium density was dose-dependently decreased with increasing S-8 concentration (Fig. 2).

### Treatment with S-8 disrupts preformed biofilm

**Figure 3:**
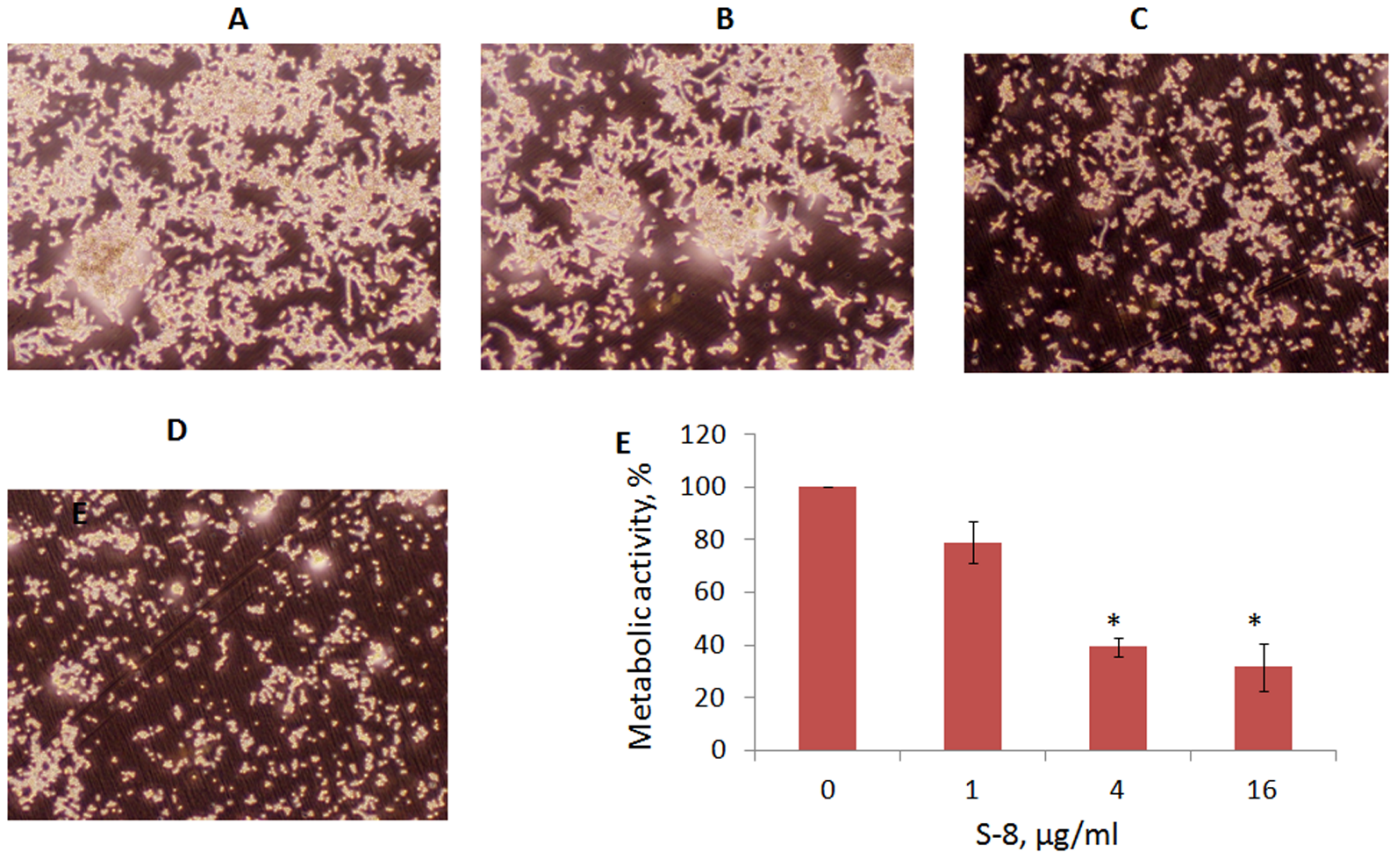
Effect of treatment with S-8 on maintenance of preformed biofilms. *C. albicans* biofilms were formed for 24 h followed by 24-h treatment with 1, 4 and 16 µg/ml S-8. Morphology of *C. albicans* in pre-formed biofilms exposed to S-8 was visualized using phase-contrast microscopy. A. Untreated control. B, C and D. Preformed biofilms exposed to 1, 4 and 16 µg/ml S-8, respectively. Magnification: 100X. At least four random fields were observed. Three independent experiments were performed. E. Biofilm metabolic activity was estimated using the XTT reduction assay. The XTT values of the untreated control were set to 100%. The results are presented as means ± SD of three independent experiments. *Significantly lower than the value for the untreated control (*P*<0.05).

**Figure 4:**
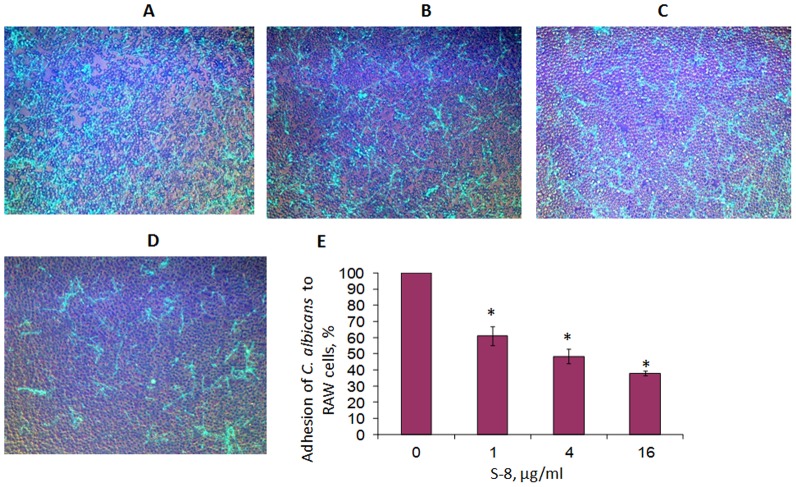
Effect of treatment with S-8 on adherence of *C. albicans* cells to RAW macrophages. A–D. *C. albicans*–GFP adhered to RAW cells in the presence or absence of S-8. A. Untreated control. B, C and D. Samples exposed to 1, 4 and 16 µg/ml S-8, respectively. Magnification: 100X. Image processing was performed using fluorescence microscopy at excitation and emission wavelengths of 488 and 522 nm, respectively. E. Quantitative analysis of fluorescence images. A value of 100% was assigned to *C. albicans* adhered to RAW cells not treated with S-8. *Significantly lower than the value for the untreated control (*P*<0.05). At least four random fields were observed and analyzed. Assays were performed in triplicate and the means ± SD from three independent experiments were calculated.

Preformed *C. albicans* biofilm was significantly attenuated by the presence of S-8 (Fig. 3). At concentrations of 1 µg/ml (Fig. 3B), 4 µg/ml (Fig. 3C) and 16 µg/ml (Fig. 3D), S-8 caused the detachment of preformed biofilms in a dose-dependent manner as compared to the control (Fig. 3A). In addition, it dramatically decreased candidal filamentation and branching in a dose-dependent manner (Fig. 3A–D). Quantitative analysis using XTT assay, in accordance with microscopic observations, revealed that S-8 at the above doses reduces metabolic activity in developed biofilms by 21%, 61% (*P*<0.05) and 70% (*P*<0.05), respectively, as compared to the untreated control (Fig. 4E).

### Inhibition of fungal attachment to mammalian macrophages by S-8

Since interactions between *C. albicans* and macrophages involve initial binding of the pathogenic organism to the surface of the host cell, we investigated whether S-8 interferes with this process. Fluorescence microscopy observations demonstrated a marked dose-dependent reduction in the number of *C. albicans* attached to RAW 264.7 mouse macrophages in the presence of S-8 at a concentration of 1 µg/ml (Fig. 4B), 4 µg/ml (Fig. 4C) and 16 µg/ml (Fig. 4D) as compared to the untreated control (Fig. 4A). Quantitative analysis of fluorescence images demonstrated that at the lowest tested dose of 1 µg/ml, S-8 had significantly decreased fungal binding to RAW cells, by 39% (*P*<0.05) compared to control. Further elevation of S-8 at concentrations of 4 µg/ml and 16 µg/ml enhanced its antiadhesion effect up to 63% (*P*<0.05), as compared to untreated controls (Fig. 4E). Neither *C. albicans* nor S-8, alone or in combination, reduced the viability of epithelial cells as determined by 3-(4,5-dimethylthiazol-2-yl)-2,5-diphenyl-tetrazolium bromide (MTT) assay (data not shown).

### Effect of S-8 on cell-surface hydrophobicity of *C. albicans*

To gain further insight into the mechanism by which S-8 treatment reduces *C. albicans* biofilm formation, we investigated whether S-8 can modify the cell-surface hydrophobicity of *C. albicans*. The hydrophobicity index (HI) was significantly and dose-dependently decreased after a 30-min treatment of *C. albicans* with S-8 (Table 1).

**Table 1:** Effect of S-8 on cell-surface hydrophobicity of *C. albicans*.

S-8, µg/ml	0	1	4	16
HI	77% (+/−2%)	68% (+/−4%)	37% (+/−1.7%)	21% (+/−0.15%)

The hydrophobicity index (HI) of *C. albicans* cells incubated for 30 min with S-8 at 0, 1, 4, and 16 µg/ml. Assays were performed in triplicate and repeated three times.

### Effect of S-8 on the expression of biofilm-associated genes in *C. albicans*

**Figure 5:**
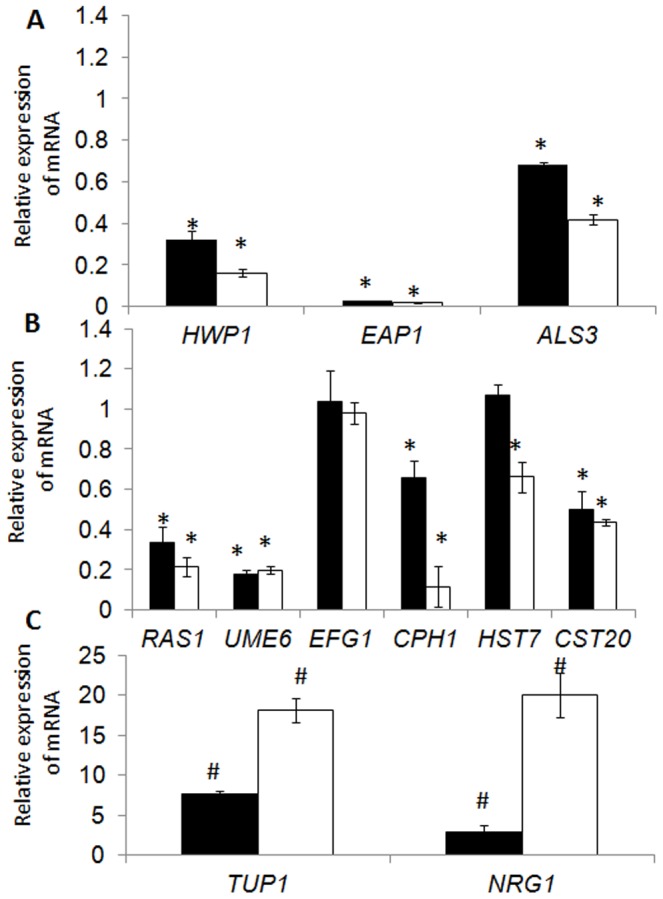
Quantitative real time RT-PCR analysis of *C*. *albicans* specific genes. *C. albicans* biofilms were formed in the presence or absence of 4 and 16 µg/ml S-8 and expression of the target genes was determined by quantitative real-time RT-PCR. Housekeeping gene *18S rRNA* was used for normalization. A. Hyphal-specific and biofilm-associated genes involved in the adhesion process. B. Hyphal transcriptional regulator genes; C. Transcriptional repressors of hyphal development. Black columns: 4 µg/ml S-8; white columns: 16 µg/ml S-8. The expression level of the untreated sample was set to 1 for each gene. *Significantly lower than the value for the untreated control (*P*<0.05). ^#^Significantly higher than the value for the untreated control (*P*<0.05). The assays were performed in triplicate and the means ± SD from three independent experiments were calculated.

To elucidate the molecular mechanism underlying S-8's inhibitory effect on adhesion properties, biofilm formation and integrity, we analyzed changes in *C. albicans* genes' expression levels in biofilms exposed to S-8 (Fig. 5). Genes involved in biofilm development were analyzed, including those associated with adhesion: *HWP1* (hyphal cell wall protein), *ALS3* (agglutinin-like sequence), *EAP1* (extracellular adhesion protein), and *UME6* (hyphal formation and maintenance. Expression of hyphal transcriptional regulators in the mitogen-activated protein kinase (MAPK) pathway (*RAS1*, *CST20*, *HST7* and *CPH1*) and the cAMP-dependent protein kinase A (PKA) pathway (*RAS1* and *EFG1*) were also assayed, as were levels of transcriptional repressors of filamentation (*TUP1* and *NRG1*). The transcriptional levels of genes involved in the adhesion process, such as the hyphal-specific genes *HWP1* and *ALS3*, as well as *EAP1*, were dose-dependently downregulated by S-8 (Fig. 5A). These genes' products are known to be associated with attachment to abiotic and mammalian cell surfaces. *EAP1* encodes a protein involved in adhesion to various surfaces, an initial and critical step in biofilm formation. We therefore consider it a biofilm-associated gene. In addition, *UME6*, a key filament-specific transcriptional regulator which is involved in long-term hyphal maintenance, was strongly suppressed by S-8 (Fig. 5B). Interestingly, expression of *EFG1—*part of the cAMP-PKA signaling cascade—was not altered by treatment with S-8, whereas three regulatory genes involved in the MAPK pathway—*CST20*, *HST7* and *CPH1*—were downregulated by this treatment (Fig. 5B). In addition, the level of *RAS1*, an upstream regulator of both pathways, was significantly decreased (Fig. 5B). Finally, transcriptional repressors of hyphal-specific gene expression, *TUP1* and *NRG1*, were dramatically upregulated by S-8 in a dose-dependent manner (Fig. 5C) which strongly correlates with alteration of candidal morphogenesis.

## Discussion

The decreasing efficacy of today's antifungal treatments and the emergence of multidrug-resistant *Candida* strains call for a new strategy in treatment perception. One venue is developing novel agents interfering with biofilm formation [24]–[26] not necessarily affecting viability of the Candida.

The TZD derivative S-8, synthesized in our laboratory, demonstrated a specific antibiofilm effect. We showed that this agent inhibited *C. albicans* biofilm formation by 50%, at a concentration eight fold lower than the MIC for planktonic cells. In addition, the tested agent disrupted developed biofilm in a dose-dependent manner. Mature biofilms are generally difficult to eradicate and are more resistant to antifungal treatments. *C. albicans* cells growing in a biofilm environment exhibit much greater resistance to antifungal agents [27], [28]. Moreover, since mature biofilms are strongly related to device-associated *Candida* infections, their elimination is critical, presenting a therapeutic challenge for management of the disease [29]. At a fourfold lower concentration than the MIC, S-8 decreased preformed biofilm by 70%, while other antifungals such as fluconazole and amphotericin B can only affect mature biofilms at doses that are 100-fold and 30-fold higher, respectively, than the MIC for planktonic cells [27], [28]. The above findings clearly indicate a specific antibiofilm effect of S-8.

Inhibition of biofilm formation by S-8 was strongly associated with alteration of fungal morphology. *C. albicans* is a dimorphic fungal pathogen which is capable of reversible transitions between yeast and hyphal forms. Morphogenesis is an important and critical determinant of *C. albicans* virulence. Strains that are defective in morphogenesis also exhibit attenuated virulence in systemic candidiasis models [30], [31]. Indeed, various studies have found that hyphal development is necessary for *C. albicans* to evade macrophages [32], escape from blood vessels [33], and colonize medical devices by forming biofilms [9], [34]. It could be that S-8 caused the detachment of hyphal form cells, and, therefore, we observed mostly firmly attached yeast cells. Numerous small molecules have been reported to regulate the yeast-to-hypha transition in *C. albicans*, and they are part of the current trend in antifungal drug development [35].

*C. albicans* can adhere to a variety of different tissues in the human body, thus facilitating its strong presence of many host niches [36]. Our study showed that S-8 significantly and dose-dependently inhibits *C. albicans* adherence to RAW macrophages. S-8's ability to inhibit *C. albicans* adherence to immune cells suggests that this compound may prevent a key and early event in *C. albicans* pathogenicity and consequently, could minimize the fungus's virulence.

Further experiments were performed to evaluate the mechanism underlying S-8's antibiofilm and antiadhesion activity. We observed a marked modification of the *C. albicans* cell surface properties from highly hydrophobic to hydrophilic following exposure to S-8. Hydrophobic interactions are generally considered to play an important role in *C. albicans* adherence to mammalian cells and also to various inert surfaces [37]. Therefore, agents that can modify the surface characteristics of *C. albicans* may alter its adherence capacity, thereby preventing biofilm formation and subsequently, invasion of the host cells [24]. Whether this effect on hydrophobicity is a surface effect, or an effect on the expression of hydrophobic proteins in the cell surface remains to be elucidated.

To test whether the antibiofilm effect of S-8 is also manifested at the transcriptional level, we tested a few different biological function genes associated with biofilm formation. The expressions of genes encoding cell-wall proteins, which play an important role in *C. albicans*' adhesion process, such as *HWP1*, *ALS3* and *EAP1*, were downregulated by S-8 (Fig. 5). *ALS* and *HWP1* have been associated with attachment to human cells and biofilm formation [9], [38], [39], while *EAP1* has been shown to play a role in adhesion to both mammalian cells and polystyrene [40]. We propose that S-8 reduced, at least in part, fungal binding to macrophages as well as *C. albicans* adhesion with subsequent biofilm formation on polystyrene plates due to a dramatic decrease in *EAP1* transcript. In addition, a marked reduction in the hyphal form after treatment with S-8 could be explained by alteration of *HWP1* expression. This observation is in accordance with a study showing that *HWP1* is not expressed during the yeast growth phase but is strongly expressed on hyphal surfaces [41]. Indeed, an *hwp1/hwp1* mutant contained exclusively yeast cells *in vitro*, while *in vivo* this mutation resulted in an absolute inability to form biofilm, indicating that the adherence of hyphae, but not of yeast, is dependent on *HWP1* expression [9]. In addition, *UME6*, which is responsible for hyphal formation and maintenance, was strongly suppressed by S-8. It has been shown that although a *ume6/ume6* mutant is able to initiate germ tubes, it is restricted in the maintenance of hyphal growth [42]. Similar results were obtained in our study: when fungal biofilm was exposed to even the lowest tested dose of S-8 (1 µg/ml), it consisted mostly of shortened filaments and the yeast form. Moreover, *UME6* is known to induce a variety of hyphal-specific transcripts, including *ALS3* and *HWP1* [43]. Therefore, S-8's interruption of *ALS3*- and *HWP1*-associated filament formation and maintenance could be attributed to downregulation of *UME6* expression. There are two major pathways regulating hyphal transformation in *C. albicans*: the MAPK (via *CPH1*) and cAMP-PKA (via *EFG1*) pathways. Three genes involved in the MAPK pathway, *CST20*, *HST7* and *CPH1*, were differently suppressed by S-8. On the other hand, although the expression level of *EFG1* was not altered by S-8, its upstream regulator and a major component of the signaling pathway of hyphal formation, *RAS1*, was markedly inhibited by the tested agent. It seems that S-8 affects both pathways. In addition, the hyphal-suppressor gene *TUP1* and the DNA-binding protein *NRG1* which functions with *TUP1* were dramatically upregulated in the presence of S-8. Studies have shown that the QS inhibitor farnesol, which inhibits filamentation [44] and biofilm formation [19] in *C. albicans*, exerts its activity by repressing both the MAPK and cAMP-PKA signaling pathways and by stimulating the expression of hyphal suppressor genes such as *TUP1* [45], [46]. *TUP1* mutants are unable to grow as yeast and instead remain locked in the filamentous form [47]. Moreover, induction of *TUP1* transcription-repressor complexes results in the downregulation of hyphal-specific gene expression [47]. These observations strongly support our hypothesis that in addition to interference with MAPK and cAMP-PKA signaling pathways, S-8 inhibits the yeast-to-hypha transition via induction of the *TUP1–NRG1* transcriptional repressor complex. In conclusion, S-8 exhibits nonfungicidal antibiofilm activity against *C. albicans* at several levels: inhibition of hyphal formation, and modification of the expression of biofilm-related functional genes and cell-surface characteristics. This alteration of fungal morphogenesis leads to impaired biofilm formation and easier eradication of a developed biofilm, as well as a reduction in the fungus's binding capacity to mammalian cells. As the TZDs are new potential antibiofilm agents, further experiments are required to evaluate the mechanism underlying S-8's antibiofilm and antiadhesion properties. Our results suggest that S-8 has potential as a promising therapeutic agent in the treatment and prevention of biofilm-associated *C. albicans* infections.

## Materials and Methods

### Synthesis of S-8

**Figure 6:**
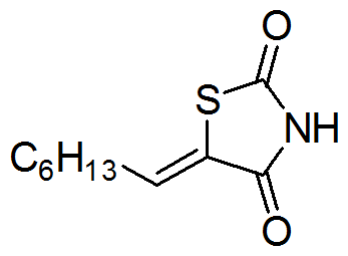
The structure of S-8.

S-8 (Fig. 6) was prepared according to a previously described method [16], [21] with some modifications. Briefly, 0.085 g (1 mmol) piperidine was added to 0.170 g (1 mmol) thiazolidine-2,4-dione in 8 ml ethanol, followed by addition of 0.098 g (1 mmol) decanal. The mixture was stirred for 30 min at room temperature then it was maintained in refluxed ethanol for 24 h. After cooling to 0°C, 1 M HCl solution was added to the mixtures and it was maintained in the refrigerator for few days. Then the precipitate was filtered, washed with petroleum ether, dried and was analyzed by NMR (File S1) and melting point to give 60% yield. The purity of the compound was above 95%.

### Fungal strains and growth conditions

*C. albicans* ATCC 90028, *C. albicans* SC5314 and *C. albicans* SC5314 carrying the GFP reporter gene (*C. albicans*–GFP) [48], kindly provided by J. Berman (Tel Aviv University, Israel), were grown for 24–48 h at 37°C on Sabouraud dextrose agar (SDA; Novamed, Jerusalem, Israel) plates. To prepare a standard cell suspension, a single colony was inoculated into YNB medium (0.67% yeast nitrogen base without amino acids, 2% dextrose) (Difco, Sparks, MD) and incubated for 18 h at 30°C with agitation. The fungal cells were harvested by centrifugation, washed twice in PBS (pH 7.4), and resuspended at 1×10^7^ cell/ml. RPMI medium (Biological Industries, Beit Haemek, Israel) supplemented with 2% glucose was used for biofilm and adhesion assays at 37°C, 5% CO_2_.

### Effect of S-8 on *C*. *albicans* biofilm formation and preformed biofilms

Fungal biofilms were prepared on commercially available, presterilized, flat-bottomed 96-well polystyrene microtiter plates (Thermo Scientific, Waltham, MA). A standard cell suspension of *C*. *albicans* ATCC 90028 (200 µl) was transferred to the wells and incubated in RPMI medium supplemented with 2% glucose in the presence of different concentrations of S-8 (0.5 µg/ml to 64 µg/ml) for 24 h at 37°C, 5% CO_2_. To investigate the effect of S-8 on preformed biofilms, *C*. *albicans* biofilms were allowed to mature for 24 h at 37°C, 5% CO_2_ as described above. The wells were washed twice with PBS, fresh RPMI medium containing sub-MICs of S-8 was added and the plate was further incubated for 24 h at 37°C, 5% CO_2_. The metabolic activity of the *C*. *albicans* biofilms as well as planktonic fungi was determined quantitatively using a standard XTT reduction assay (Biological Industries) [16]. The assays were performed in triplicate and repeated three times.

### XTT reduction assay

Prior to each assay, XTT solution (Biological Industries) was thawed and mixed with a N-methyl dibenzopyrazine methyl sulfate (PMS) (Biological Industries) solution at 50∶1 (v/v). The biofilms were washed and then incubated with 60 µl of the XTT–PMS solution in 2 ml of PBS. To determine planktonic cell viability, 60 µl of the XTT–PMS solution was added to the supernatants aspirated from the biofilms and placed in a new 96-well plate. Plates containing biofilms as well as supernatants were then incubated in the dark for 3 h at 37°C. Following incubation, the color change in the solution was measured spectrophotometrically at 492 nm using a GENios plate reader (Tecan, Salzburg, Austria).

### Effect of S-8 on *C. albicans* morphology

Fungal biofilms developed in the presence of S-8 or preformed biofilms after exposure to S-8 were washed with PBS and immediately processed for observation. Morphology of fungi in untreated and S-8-treated biofilm was visualized under an Olympus CKX41 inverted microscope (Tokyo, Japan) and photographed with a DP72 camera. Calculation of average filament length and yeast-form number in each sample was performed using Image J v3.91 software (http://rsb.info.nih.gov/ij). At least four random fields were observed. Three independent experiments were performed at different times. Assays were performed in triplicate and repeated three times.

### Effect of S-8 on adhesion of *C. albicans* to RAW macrophages

This assay was performed using the modified method of Feldman et al. [24]. Briefly, RAW 264.7 mouse macrophages were cultured in Dulbecco's Modified Eagle's Medium (DMEM) (Sigma–Aldrich, St. Louis, MO) supplemented with 10%fetal bovine serum (FBS), penicillin (1 IU/ml), streptomycin (1 µg/ml) and L-glutamine (200 mM) in a humidified incubator at 37°C with 5% CO_2_ until 80% confluence. The cells were seeded at a concentration of 4×10^5^ cell/ml under the above conditions in sterile 96-well clear-bottom black microplates (Greiner Bio One, Frickenhausen, Germany) and incubated until confluence. Then, the wells were washed with DMEM-1% heat-inactivated FBS, blocked with 1% bovine serum albumin (BSA) to prevent nonspecific fungal attachment, and treated with S-8 diluted in DMEM-1% heat-inactivated FBS medium at sub-MIC for 1 h in a 5% CO_2_ atmosphere at 37°C. Untreated control wells (no S-8) were also prepared. Subsequently, cells from an overnight culture of *C. albicans*–GFP at 10^7^ cell/ml in RPMI were applied at a multiplicity of infection (MOI) of 10 (10 *C. albicans* per mammalian cell) to S-8-pretreated or control RAW cells and incubated for 30 min at 37°C. All incubation and following washing steps were carried out in the dark. After incubation, unbound *C. albicans* was aspirated and wells were washed three times with PBS. Image processing was performed using an Olympus CKX41 inverted microscope (Tokyo, Japan) supported with fluorescence filters and photographed with a DP72 camera. Images of adhered *C. albicans*–GFP were observed at excitation and emission wavelengths of 488 and 522 nm, respectively. Image analysis was performed using Image J v3.91 software (http://rsb.info.nih.gov/ij). Three high-power fields (100X) were selected for analysis of each tested condition. The assays were performed in triplicate and repeated three times.

### Evaluation of S-8 and *C. albicans* cytotoxicity

RAW cells were cultured as described above. For cytotoxicity assay, mammalian cells (2×10^4^ cells; 100 µl) were seeded in 96-well microtiter plates for 24 h, then treated with increasing concentrations of S-8 (0, 1, and 16 µg/ml), with *C. albicans* at a MOI of 10, or with both S-8 and *C. albicans*. Cell viability was determined by MTT assay (Roche Diagnostics, Mannheim, Germany) after 24 h. MTT solution (5 mg/ml) was prepared by dissolving MTT powder in PBS, and filter-sterilizing (0.22 µm pore-size filter) to remove insoluble residue. At the end of the incubation, the supernatant was aspirated and MTT solution (20 µl) was added to each well and incubated for 4 h at 37°C. The solution was replaced with 1 ml DMSO (Sigma–Aldrich) to dissolve the dark blue formazan crystals. After incubation for 15 min at room temperature, the absorbance was read in a GENios plate reader at 570 nm. The assays were performed in triplicate and repeated three times.

### Effect of S-8 on *C. albicans* cell-surface hydrophobicity

This assay was performed according to the method described by Feldman et al. [24], using xylene as the organic solvent. Briefly, *C. albicans* ATCC 90028 at a concentration of 10^7^ cell/ml was incubated for 30 min at 37°C with S-8 at 0, 1, 4, and 16 µg/ml. Fungal cells were then washed with PBS, suspended in the same buffer, and the optical density was determined spectrophotometrically at 660 nm. The cells were mixed with xylene (2.5∶1, v/v), shaken for 2 min, and the tube was left for 20 min at room temperature for phase separation. The turbidity of the aqueous phase was read at 660 nm. The hydrophobicity index was calculated as HI  =  (OD_control_ - OD_test_) × 100/OD_control_, where OD_control_  =  optical density at 660 nm before xylene treatment and OD_test_  =  optical density at 660 nm after xylene treatment. Assays were performed in triplicate and repeated three times.

### Quantitative real time RT-PCR analysis of *C*. *albicans* specific genes

Biofilms of *C. albicans* SC5314 were grown in the presence of S-8 in 6-well plates under the conditions described above. After washing with PBS, biofilm cells were removed from the bottom of the plates with a sterile scraper following disruption in a Fast Prep Cell Disrupter (Bio 101, Savant Instruments, Inc., NY, USA). Total RNA was extracted from fungal biofilms using Tri-Reagent (Sigma–Aldrich) by a previously described method [19]. RNA concentration was determined spectrophotometrically using a Nanodrop ND-1000 Instrument (Wilmington, DE, USA). 2 µg of template was reverse-transcribed with Super Script First Strand (Invitrogen, Life Technologies, Carlsbad, CA, USA). The integrity and purity of the RNA was assessed using an Agilent 2100 Bioanalyzer system (Agilent Technologies, Santa Clara, CA, USA). Expression of hyphal-specific and biofilm-associated genes (*ALS3*, *EAP1*, *HWP1*) and their transcriptional regulators (*RAS1, UME6, CPH1, CST20, HST7, EFG1*, *TUP1, NRG1*) was analyzed. The relative expression levels of the target genes were analyzed using an ABI-Prism 7300 Instrument (Applied Biosystems, Foster City, CA, USA). Platinum SYBR Green PCR Master Mix (Invitrogen) was used to monitor the amplified product in real time, following the manufacturer's protocol. Primers for the tested genes were taken from the literature (Table S1). For each set of primers, a standard amplification curve (critical threshold cycle vs. exponential of concentration) was plotted, and only those with slope ≈ -3 were considered reliable. The PCR consisted of denaturation at 95°C for 10 min, followed by 40 cycles of amplification (95°C for 10 s, 55°C for 10 s, and 72°C for 10 s) and quantification. The expression of 18S *rRNA* was used for normalization and to calculate the relative changes in target gene expression. Control reactions were also performed with RNA that had not been reverse-transcribed to ensure that no genomic DNA was amplified during the PCRs. Gene expression is given in relative values, setting the expression level of the untreated control to 1 for each gene. The assays were performed in triplicate and repeated three times.

### Statistical analysis

Means ± standard deviation were calculated. The statistical analysis was performed using Student's t-test with a significance level of *P*<0.05.

## Supporting Information

File S1 (DOC)

Table S1 (DOCX)
