# Supplementary material for: Therapeutic Potential of Thiazolidinedione-8 as an Antibiofilm Agent against Candida albicans
Source: PLoS One. 2014 May 5;9(5):e93225. doi: 10.1371/journal.pone.0093225 (PMC4010395; doi:10.1371/journal.pone.0093225)
Supplement: File S1 — (DOC) [file pone.0093225.s001.doc]

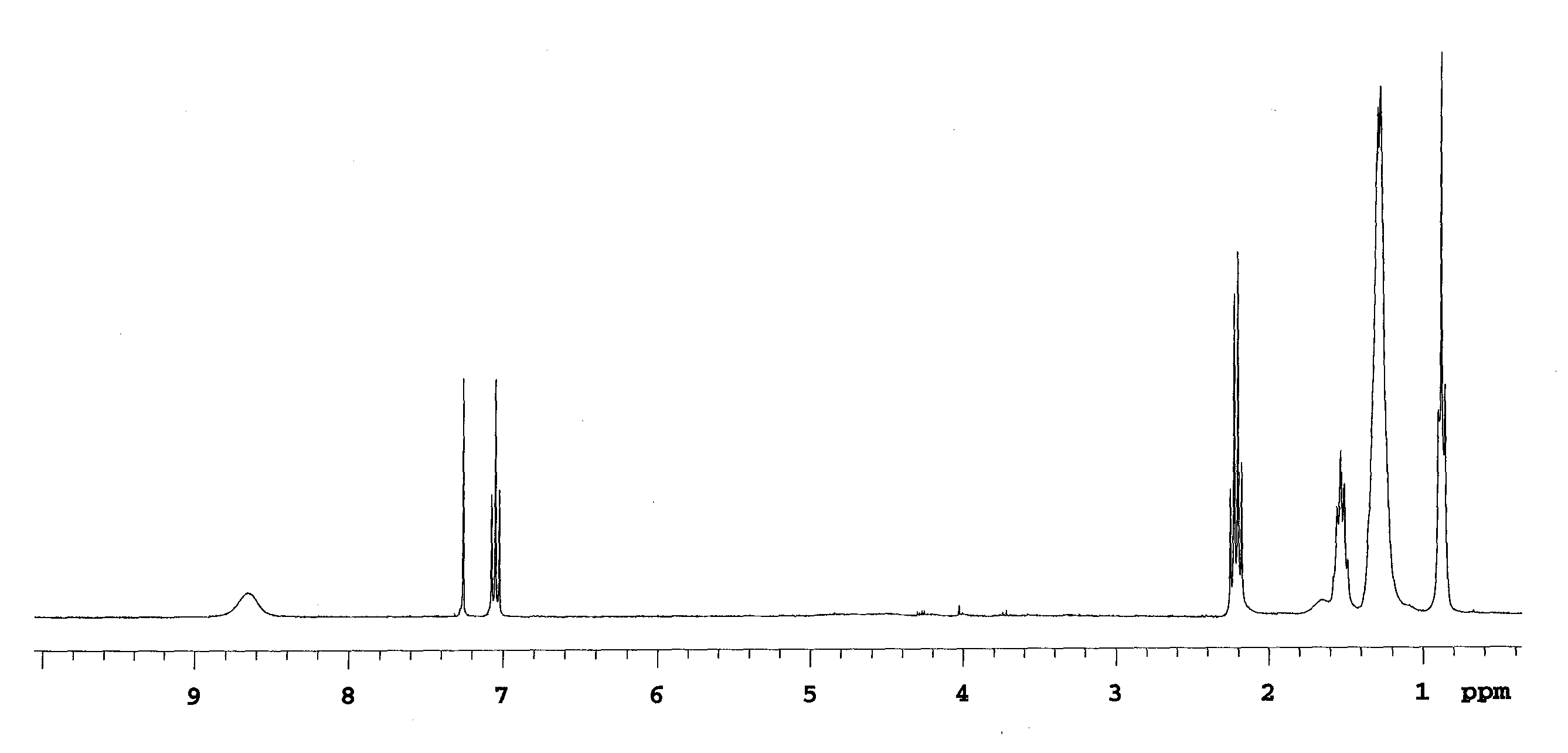
^1^H NMR (300 MHz, Chloroform d): δ 0.85 (t, 3H, *J*_HH_ = 7.2 Hz), 1.20-1.40 (overlap, 6H), 1.57 (m, 2H), 2.21 (m, 2H), 7.07 (t, 1H, *J*_HH_ = 8.4 Hz), 8.63 (broad s, 1H); ^13^C NMR (75.5 MHz, Chloroform d): δ14.07, 22.56, 27.78, 28.91, 29.12, 31.62, 31.82, 126.18, 139.88, 165.80, 167.50; Anal. Calcd for C_11_H_17_NS: C, 58.12; H, 7.54; N, 6.16; S, 14.11. Found: C, 57.91; H, 7.47; N, 6.27; S, 14.29; Melting point: 71 ^0^C.

^1^H NMR (300 MHz, Chloroform d) of S-8


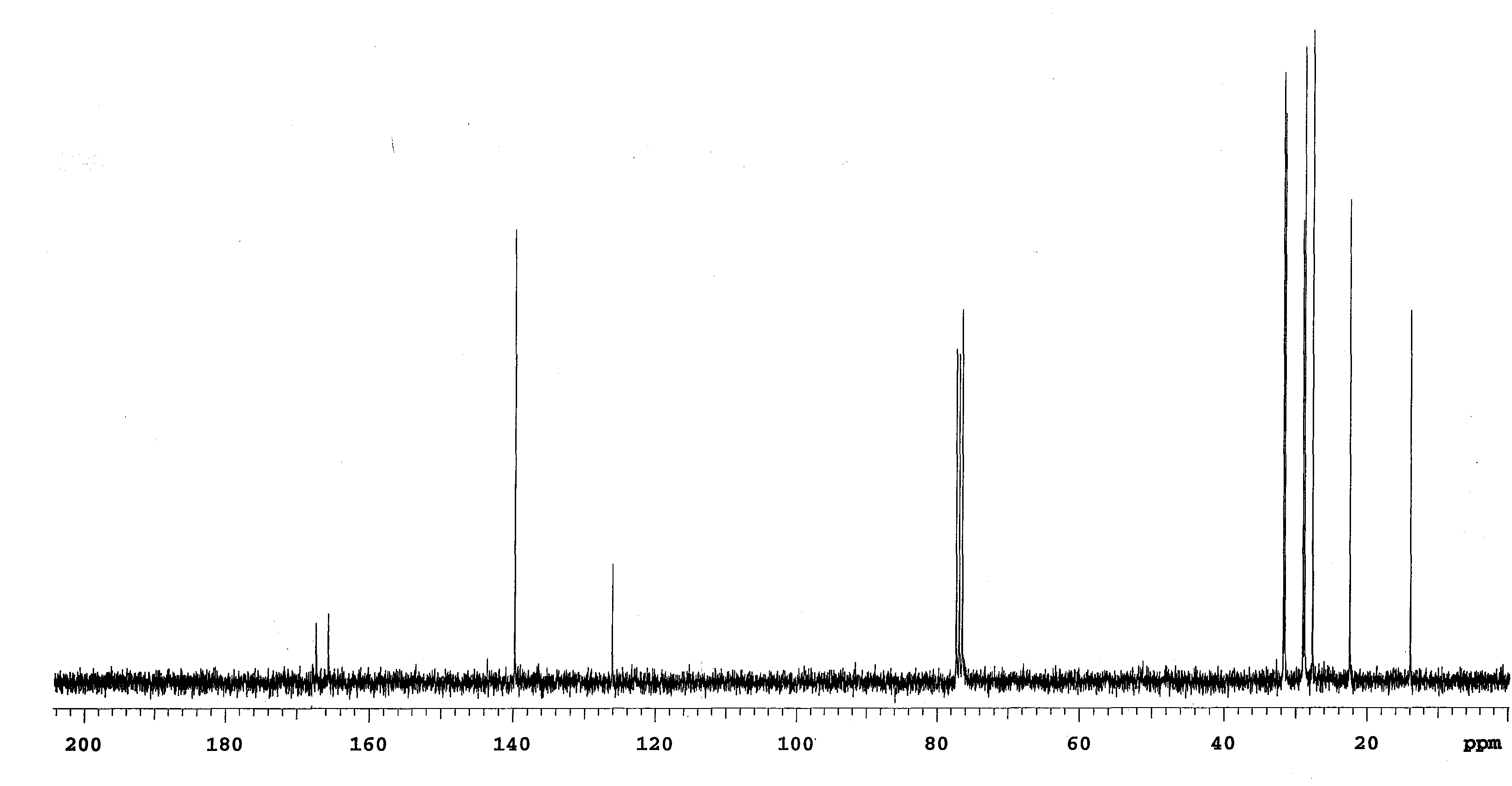


^13^C NMR (75.5 MHz, Chloroform d) of S-8
