# Supplementary material for: Therapeutic Potential of Thiazolidinedione-8 as an Antibiofilm Agent against Candida albicans
Source: PLoS One. 2014 May 5;9(5):e93225. doi: 10.1371/journal.pone.0093225 (PMC4010395; doi:10.1371/journal.pone.0093225)
Supplement: Table S1 — (DOCX) [file pone.0093225.s002.docx]

| **Supplementary Table S2. Primers used in this study Primer name** | **Primer sequence (5′–3′)** |
| --- | --- |
| 18S rRNA-F | CACGACGGAGTTTCACAAGA [1] |
| 18S rRNA-R | CGATGGAAGTTTGAGGCAAT [1] |
| ALS3-F | CAACTTGGGTTATTGAAACAAAAACA [2] |
| ALS3-R | AGAAACAGAAACCCAAGAACAACC [2] |
| EFG1-F | GCCTCGAGCACTTCCACTGT [2] |
| EFG1-R | TTTTTTCATCTTCCCACATGGTAGT [2] |
| EAP1-F | TGTGATGGCGGTTCTTGTTC [3] |
| EAP1-R | GGTAGTGACGGTGATGATAGTGACA [3] |
| CPH1- F | TATGACGCTTCTGGGTTTCC [4] |
| CPH1- R | ATCCCATGGCAATTTGTTGT [4] |
| CST20- F | ATGTCTCATAATAATGGC [5] |
| CST20- R | GGTTAATTAGTTTCTTC [5] |
| NRG1-F | CCAAGTACCTCCACCAGCAT [6] |
| NRG1-R | GGGAGTTGGCCAGTAAATCA [6] |
| TUP1-F | CTTGGAGTTGGCCCATAGAA [6] |
| TUP1-R | TGGTGCCACAATCTGTTGTT [6] |
| HWP1F | GCTCCTGCTCCTGAAATGAC [6] |
| HWP1R | CTGGAGCAATTGGTGAGGTT [6] |
| RAS1-F | CCCAACTATTGAGGATTCTTATCGTAAA [7] |
| RAS1-R | TCTCATGGCCAGATATTCTTCTTG [7] |
| UME6-F | ACCACCACTACCACCACCAC [8] |
| UME6-R | TATCCCCATTTCCAAGTCCA [8] |
| HST7-F | TCATCAGCTTCTTCTATAC [9] |
| HST7-R | TATTGAGGAAATGACAGTT [9] |

1. Morschhäuser J, Barker KS, Liu TT, BlaB-Warmuth J, Homayouni R, Rogers PD. (2007) The transcription factor Mrr1p controls expression of the MDR1 efflux pump and mediates multidrug resistance in *Candida albicans*.PLoS Pathog. 3:e164.

2. Uppuluri P, Dinakaran H, Thomas DP, Chaturvedi AK, Lopez-Ribot JL. (2009) Characteristics of *Candida albicans* biofilms grown in a synthetic urine medium.

J Clin Microbiol 47:4078-83

3. Samaranayake YH, Cheung BP, Yau JY, Yeung SK, Samaranayake LP. (2013) Human serum promotes *Candida albicans* biofilm growth and virulence gene expression on silicone biomaterial. PLoS One 8:e62902

4. Ramírez-Zavala B, Weyler M, Gildor T, Schmauch C, Kornitzer D, Arkowitz R, Morschhäuser J. (2013) Activation of the Cph1-dependent MAP Kinase signaling pathway induces white-opaque switching in *Candida albicans*. PLoS Pathog. 9:e1003696.

5. Cheng S, Clancy CJ, Checkley MA, Handfield M, Hillman JD, Progulske-Fox A, Lewin AS, Fidel PL, Nguyen MH. (2003) Identification of *Candida albicans* genes induced during thrush offers insight into pathogenesis.Mol Microbiol 48:1275-88.

6. Holcombe LJ, McAlester G, Munro CA, Enjalbert B, Brown AJ, Gow NA, Ding C, Butler G, O'Gara F, Morrissey JP. (2010) *Pseudomonas aeruginosa* secreted factors impair biofilm development in *Candida albicans*. Microbiol 156:1476-86.

7. Tsang PW-K, Bandara HMHN, Fong W-P (2012) Purpurin Suppresses *Candida albicans* Biofilm Formation and Hyphal Development. PLoS One 7: e50866.

8. O'Connor L, Caplice N, Coleman DC, Sullivan DJ, Moran GP. (2010) Differential filamentation of *Candida albicans* and *Candida dubliniensis* is governed by nutrient regulation of UME6 expression.Eukaryot Cell. 9:1383-97.

9. Murzyn A, Krasowska A, Stefanowicz P, Dziadkowiec D, Łukaszewicz M. (2010) Capric acid secreted by *S. boulardii* inhibits *C. albicans* filamentous growth, adhesion and biofilm formation.PLoS One. 5:e12050.
